# Tackling the hard problems: implementation experience and lessons learned in newborn health from the African Health Initiative

**DOI:** 10.1186/s12913-017-2659-4

**Published:** 2017-12-21

**Authors:** Hema Magge, Roma Chilengi, Elizabeth F. Jackson, Bradley H. Wagenaar, Almamy Malick Kante, the AHI PHIT Partnership Collaborative, Ahmed Hingora, Dominic Mboya, Amon Exavery, Kassimu Tani, Fatuma Manzi, Senga Pemba, James Phillips, Almamy Malick Kante, Kate Ramsey, Colin Baynes, John Koku Awoonor-Williams, Ayaga Bawah, Belinda Afriyie Nimako, Nicholas Kanlisi, Elizabeth F. Jackson, Mallory C. Sheff, Pearl Kyei, Patrick O. Asuming, Adriana Biney, Roma Chilengi, Helen Ayles, Moses Mwanza, Cindy Chirwa, Jeffrey Stringer, Mary Mulenga, Dennis Musatwe, Masoso Chisala, Michael Lemba, Wilbroad Mutale, Peter Drobac, Felix Cyamatare Rwabukwisi, Lisa R. Hirschhorn, Agnes Binagwaho, Neil Gupta, Fulgence Nkikabahizi, Anatole Manzi, Jeanine Condo, Didi Bertrand Farmer, Bethany Hedt-Gauthier, Kenneth Sherr, Fatima Cuembelo, Catherine Michel, Sarah Gimbel, Bradley Wagenaar, Catherine Henley, Marina Kariaganis, João Luis Manuel, Manuel Napua, Alusio Pio

**Affiliations:** 1 Division of Global Health Equity, Brigham and Women’s Hospital, 75 Francis Street, Boston, MA 02115 USA; 2 Division of General Pediatrics, Boston Children’s Hospital, Boston, MA USA; 3 Partners In Health, Kigali, Rwanda; 4 Partners In Health, Boston, MA USA; 5 Centre for Infectious Disease Research in Zambia, Lusaka, Zambia; 6 Heilbrunn Department of Population and Family Health, Mailman School of Public Health, Columbia University, New York, NY USA; 7 Department of Global Health, University of Washington, Seattle, WA USA; 8 Health Alliance International, Seattle, WA USA; 9 Bloomberg School of Public Health, Johns Hopkins University, Baltimore, MD USA

**Keywords:** Newborn health, Maternal child health, Health system strengthening, Quality of care, Neonatal mortality, Ghana, Mozambique, Rwanda, Tanzania, Zambia

## Abstract

**Background:**

The Doris Duke Charitable Foundation’s African Health Initiative supported the implementation of Population Health Implementation and Training (PHIT) Partnership health system strengthening interventions in designated areas of five countries: Ghana, Mozambique, Rwanda, Tanzania, and Zambia. All PHIT programs included health system strengthening interventions with child health outcomes from the outset, but all increasingly recognized the need to increase focus to improve health and outcomes in the first month of life. This paper uses a case study approach to describe interventions implemented in newborn health, compare approaches, and identify lessons learned across the programs’ collective implementation experience.

**Methods:**

Case studies were built using quantitative and qualitative methods, applying the World Health Organization Health Systems Strengthening Framework, and maternal, newborn and child health continuum of care framework. We identified the following five primary themes in health systems strengthening intervention strategies used to target improvement in newborn health, which were incorporated by all PHIT projects with varying results: health service delivery at the community level (Tanzania), combining community and health facility level interventions (Zambia), participatory information feedback and clinical training (Ghana), performance review and enhancement (Mozambique), and integrated clinical and system-level improvement (Rwanda), and used individual case studies to illustrate each of these themes.

**Results:**

Tanzania and Zambia included significant community-based components, including mobilization and sensitization for increased uptake of essential services, while Ghana, Mozambique, and Rwanda focused more efforts on improving the quality of services delivered once a patient enters a health facility. All countries included aspects that improved communication across levels of the health system, whether through district-wide data sharing and peer learning networks in Mozambique and Rwanda, or improved referral processes and systems in Tanzania, Zambia, and Ghana.

**Conclusion:**

Key lessons learned include the importance of focusing intervention components on addressing drivers of neonatal mortality across the maternal and newborn care continuum at all levels of the health system, matching efforts to improve service utilization with provision of high quality facility-based services, and the critical role of leadership to catalyze improvements in newborn health.

## Background

While child mortality has improved worldwide, catalyzing similar advances during the newborn period has proven more challenging [1]. Key interventions such as immunizations, water and sanitation, integrated management of childhood illness (IMCI), and HIV/AIDS programs have contributed to these significant advances in child health; however, they have not been met with the same degree of progress during the first month of life; neonatal mortality now contributes to over 40% of deaths under the age of five, and complications related to prematurity have risen to the top cause of childhood deaths [2].

Despite numerous evidence-based interventions to improve newborn survival, coverage and quality remain significant challenges to accelerating change [3]. Drivers of neonatal mortality occur along multiple points in time – from the antenatal, to birth, to postnatal periods – and at all levels of the health system, from communities to hospitals. Addressing the key causes of neonatal mortality such as sepsis, birth asphyxia, and prematurity requires high functioning and responsive health systems that can deliver quality routine and emergency care in a timely manner. Critical bottlenecks in key system building blocks affecting newborn health include: health worker shortages (such as nurses, midwives, and specialists, anesthetists), clinical skills gaps in management of routine and complex care, absence or stock-out of essential commodities and supplies, gaps in the use of data for learning and decision-making, weak leadership and priority setting, and lack of adequate resource allocation. Therefore, strengthening health delivery systems to deliver improved quality of care and improve health seeking for quality services across this continuum of maternal, newborn, and child health processes is critical to achieving progress in neonatal mortality [1, 3–5].

The Doris Duke Charitable Foundation’s African Health Initiative (AHI) created a platform for implementation and rigorous evaluation of health system strengthening interventions to improve population health in selected areas of five countries: Ghana, Mozambique, Rwanda, Tanzania, and Zambia. All country programs included were implemented in politically stable settings, a mix of rural and urban settings, and with a broad goal of reducing mortality among children under 5. Within the first few years of broader health systems strengthening, all of the Population Health Implementation and Training (PHIT) Partnership teams recognized that neonatal mortality was representing a larger proportion of under-5 deaths in their countries and in their implementation areas specifically, and so initiated or strengthened strategies designed to improve neonatal health outcomes. We used case study methodology to retrospectively describe the design and implementation of the neonatal-focused interventions to compare approaches, and identify lessons learned across the collective experience.

## Methods

Each country program team designed their broader PHIT intervention following the World Health Organization (WHO) building block framework [6]. Case study methodology was used to describe and analyze aspects of the health system strengthening interventions implemented in each country that related to neonatal health and outcomes using the WHO building blocks framework, and aspects that were targets of interventions to address key steps in the maternal and newborn health continuum of care framework. This continuum is defined as ensuring quality care from pregnancy through delivery and immediate and later postnatal care [5].

Quantitative and qualitative methods were used to build each case in its country context. Each PHIT team chose a representative to lead their participation. Indicators related to maternal and newborn health used in all PHIT programs were identified and collected by these representatives from routinely and non-routinely collected programmatic data. These data were reviewed and compared to contextualize each program case study. Representatives also had the option of including additional program-specific quantitative data relevant to their intervention implementation (see Tables 1, 2, 3, 4 and 5). Specific methods used for each project table are described in consequent relevant country sections.

Qualitative semi-structured surveys followed by key informant interviews with additional leaders of each PHIT team were used to build each case supplemented by targeted review of results already published by the teams. Each of the five team representatives responded to a semi-structured survey of closed and open-ended questions with support from other team members as needed. After survey completion, key informant interviews were conducted with two to three additional members of each PHIT team to enrich survey data. Country program cases were then developed and reviewed with the program representatives.

**Fig. 1:**
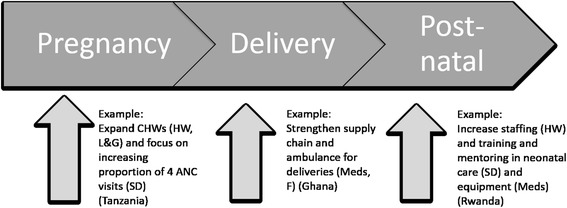
Examples of health systems strengthening activities targeting reductions in neonatal mortality addressing one or more of six WHO building blocks: service delivery (SD), health workforce (HW), essential medicines (and equipment) (Meds), health information systems (HIS), financing (F), and leadership and governance (L&G)

Case studies were developed and analyzed to identify components of the intervention related to each WHO building block that were explicitly (neonatal care focused) or implicitly (broad health system strengthening or maternal and child health) related to neonatal health. Interventions were also analyzed to identify key processes targeting specific steps along the continuum of care lifecycle approach to newborn health (reproductive health, antenatal care, delivery management, and postnatal care) [5] (see Fig. 1). Inductive methods were used to capture emerging themes of interventions and implementation following the two frameworks, and each case study was used to describe one predominant theme in more detail. A cross-case analysis also was used to identify commonalities, differences, and lessons learned across case studies.

## Results

### Baseline characteristics and context

Baseline data were collected at the start of the African Health Initiative in all five PHIT countries using agreed-upon common indicators [7]. (Descriptive programmatic data related to newborn health is shown in Table 6.) All settings had skilled delivery rates over 50%, but variable utilization of antenatal and postnatal care. All settings had high rates of at least one antenatal care (ANC) visit, but all, with the exception of the Ghana PHIT project, had low rates of four or more ANC visits. Postnatal care coverage was low across all countries.

All country PHIT programs focused on health system improvement approaches designed to address the gaps identified in their local setting [4, 8–12]. We identified the following five primary themes in the overall health system intervention strategies which covered, indirectly or explicitly, improvement of health services for newborns and reduction of preventable deaths: 1) health service delivery at the community level, 2) combining community and health facility level interventions, 3) participatory information feedback and clinical training, 4) performance review and enhancement, and 5) integrated clinical and system-level improvement. We describe country case studies highlighting each theme below as they relate to the work to reduce neonatal mortality. Some country projects added or intensified their focus on neonatal mortality in the course of their implementation timeline (Zambia, Rwanda), while others integrated strong neonatal focus from the outset (Tanzania, Ghana, Mozambique). Data on final overall impact on neonatal mortality in the projects was not available at the time of this study.

### A community-based health system strengthening approach to increase newborn health services: Tanzania Connect

#### Implementation objectives

The PHIT Partnership in Tanzania (Connect) was designed as a randomized cluster trial in 2011 to measure the impact of posting paid community health workers, named community health agents (CHAs), on child mortality rates in three rural districts of Tanzania (Rufiji, Kilombero, and Ulanga) [10]. This was based on the goal of supporting the government of Tanzania’s policy for every village to have a health facility.

#### Interventions

To improve access to maternal and newborn health services, Connect implemented a number of strategies, including community-based service provision, improved case detection and referrals, support for telecommunication, vehicle support, and data feedback. First, CHAs were trained for 9 months to enable them to provide a number of key services. CHAs in communities conducted home visits during pregnancy to counsel on and promote early initiation of ANC within the first 4 months of pregnancy and at least four subsequent ANC visits, and to discuss birth preparedness and the importance of facility-based deliveries. When deliveries occurred at home, CHAs coordinated immediate facility assessment. CHAs were tasked with conducting at least four postnatal home visits starting 24 hours after delivery, followed by visits at three, seven, and 28 days after delivery. The CHAs’ main tasks during these visits were to identify mothers and newborns with complications and refer them to the nearest health facility; provide intensive counseling on essential newborn care, including immunizations and exclusive breastfeeding; screen for low birth weight; and educate households on maternal and newborn postpartum danger signs.

Finally, the Connect team organized a one-day annual meeting to share results with CHAs and their supervisors (nurses and midwives), as well as representatives from the District Medical Office. By the 2013 annual meeting, results had shown that the intervention had not led to changes in neonatal mortality rates. Therefore, from that point forward, improving the use of the emergency referral systems for sick newborns immediately to health facilities was prioritized. Connect complemented this community-based referral process with additional investments, including: 1) extending the provision of cell phones to all health facilities (CHAs and their supervisors had this service before) with unlimited calls to a closed user group for emergency referrals; 2) repairing six ambulances in Kilombero and Ulanga districts; 3) purchasing a vehicle ambulance and a boat ambulance for Rufiji district, and; 4) training health workers and ambulance drivers in first aid.

#### Evaluation methods

Table 1 shows coverage and behavioral indicators before and after the CHAs were deployed in August 2011. Identical cross-sectional household surveys were conducted at baseline (2011) and endline (2015) on the same household sample with as many of the same field interviewers as possible. A list of all households (sampling frame), from which 2183 were successfully interviewed in 2011, was obtained from the surveillance population, and a probability proportional to size (PPS) sampling technique was used to select the sample. During the 2015 survey, only five households had dissolved and other respondents could not be located. Ultimately, the endline successfully visited 2178 households.

**Table 1:** Baseline coverage of healthcare worker practice indicators August 2009–July 2011, Rufiji, Ulanga, and Kilombero Districts, Tanzania^a^

Indicator	Number (%)
C-Section	117/915 (12.8)
Facility based delivery	682/915 (74.5)
Essential newborn care (wrapping newborn before the placenta delivery)	693/889 (78.0)
Essential newborn care (wiping/drying newborn before the placenta was delivery)	640/889 (72.0)
Immediate breastfeeding (within 1 h after birth)	453/889 (51.0)
Postnatal care visit for newborn only (at least 1)	540/889 (60.7)
Postnatal care visit for mother only (at least 1)	274/889 (30.8)
Postnatal care visit for newborn only by CHW (at least 1)	74/889 (8.3)
Postnatal care visit for mother only by CHW (at least 1)	48/889 (5.4)

^a^Data from Baseline Household Survey, 2011

#### Implementation outcomes

Health improvements have been seen in both the intervention and comparison groups. Despite the improvement seen, the intervention did not appear to have any additional effect in terms of antenatal care utilization of four or more visits, use of a skilled attendant at birth, or postnatal care utilization at the health facility and community levels for both the mother and the newborn, indicating that improvements were unlikely to have been related to the interventions implemented by the Connect project. Additional qualitative study is underway to explore potential reasons for this lack of impact.

Challenges encountered in the program included delays in payment of the CHAs’ monthly salary, which affected their motivation and activity level; shortage of supplies and supervision activities, and; poor quality of care at facilities when referrals by CHAs were achieved, since the project did not improve the quality of service at the facility level.

### Combining community and facility-based approaches to newborn care: Better Health Outcomes through Mentoring and Assessment in three districts of rural Zambia

#### Implementation objectives

The PHIT project in Zambia (Better Health Outcomes through Mentoring and Assessment, or BHOMA) implemented a complex quality improvement program in 42 rural health facilities in the districts of Chongwe, Kafue, and Luangwa in Lusaka province beginning in 2011. The detailed methodology of BHOMA has been reported elsewhere [12] and included the deployment of quality improvement teams to implement clinical protocols, define clinical quality expectations, and provide tools to deliver on these expectations at each site. After initially focusing on general outpatient care, the project team found little evidence from their quality of care data to indicate improvement in neonatal care. Therefore, the team ramped up efforts to target improvements in maternal and newborn care in the last year of the project, increasing demand for complete antenatal care attendance and health facility deliveries, and providing improved quality of care at facilities.

#### Interventions

Project efforts at the community level worked to improve health-seeking among pregnant women through community mobilization. Community volunteers conducted home visits to pregnant women, during which they discussed birth preparedness and the importance of facility-based deliveries. Traditional birth attendants (TBAs) were provided with register books to report contact with pregnant women and pregnancy outcomes for women in the catchment areas. TBAs were trained to identify women having home deliveries and escort them to the nearest health facility as soon as possible. The TBAs also conducted postnatal home visits at 2 days of life for every newborn within their communities. The main activities during these visits were to screen for neonatal danger signs and to encourage the mothers to attend facility-based postnatal clinics. Additionally, in each village, community-based Safe Motherhood Action Groups were trained to provide continuous sensitization activities around key maternal and newborn health topics.

At the health facility level, the BHOMA intervention focused on strategies to improve quality of antenatal, delivery, and postnatal care. This included the initial supply of key equipment to ensure that providers had the necessary tools to provide quality care, training, and mentoring.

In ANC, providers were trained to counsel pregnant women on key health topics, including the importance of at least four ANC visits, facility-based delivery, and prevention of infectious diseases, such as malaria, HIV, and syphilis. To improve delivery management, simplified partographs were introduced and facility health care providers were trained in essential maternal and newborn care. Furthermore, PHIT program staff conducted onsite mentoring and supervisory visits at the delivery sites to assess the adherence to guidelines for managing the normal births as well as those with common complications. From these chart reviews, areas of weak performance were identified and retraining was undertaken accordingly. Mentoring visits were jointly conducted with District Medical Office teams with supervision plans integrated into annual district plans to support integration of the program into the public system and ongoing sustainability beyond the BHOMA project period.

To improve postnatal facility-based care, a “sick” neonatal form was designed to augment the existing pediatric form to address specific neonatal illnesses and facilitate clinical decision-making. Furthermore, a week one visit encounter data point was added to data monitoring to track early health system contact with the neonate and facilitate efforts to improve coverage of postnatal care.

#### Evaluation methods

Across all system levels, facilitating data collection and use was also a core intervention. Community health workers were provided with special phones and trained on tracking and completing follow up data collection using these phones [13]. Touch screen computers and data servers were installed at each of the facilities to provide access to an electronic medical record system, which was linked in real time to the district medical office and the central PHIT project office in Lusaka.

Between 2013 and 2015, using PHIT-supported cross-sectional surveys, steady improvements were recorded in key indicators related to newborn health, including ANC attendance, at least four ANC visits, use of modern contraceptives, and health facility deliveries (Table 2).

**Table 2:** Improvements in maternal newborn care processes in Zambia^1^

Indicator	Number and prevalence 2013	Number and prevalence 2015	Absolute percent change
ANC any attendance	9340/10,152 (92%)	10,165/10,351 (98.2%)	6.8
ANC 4 visits	5096/10,152 (50.2%)	6335/10,351 (61.2%)	11
Use of any modern contraceptive method	15,608/48,322 (32.3%)	19,597/51,302 (38.2%)	5.9
Health facility deliveries	4264/10,152 (42%)	5434/10,531 (52.5%)	10.5

^1^Data from project cross sectional community Surveys 2013 and 2015

#### Implementation outcomes

Several challenges were experienced in the BHOMA project. A critical shortage of trained midwives led to TBAs attending deliveries even at health facilities despite policy directives to focus their activities in the community. Additionally, a lack of appropriate maternal newborn care services was frequent with shortages of reagents for routine tests in pregnancy. The team encountered difficulties with ascertainment of population numbers, especially with regard to population migrations for the catchment areas of the intervention sites. Finally, pooling of data from clinic records, TBA registers, and cross-sectional community surveys to generate a comprehensive picture was challenging given that the routine data was not sufficient for tracking the required indicators for the program.

### Participatory information feedback and clinical training: Ghana Essential Health Intervention Program in Ghana’s Upper East Region

#### Implementation objectives

The PHIT Ghana Essential Health Intervention Program (GEHIP) was designed to increase universal access to health care by expanding coverage of the national primary health care system, called Community-Based Health Planning and Services (CHPS) [14], at the community level in Ghana’s Upper East Region. Prior to GEHIP, CHPS had transformed community level health care in Ghana by relocating nurses from static facilities to reside and work at the village and household level. CHPS reduced maternal mortality by over 40% and total fertility by one birth, achieved the Millennium Development Goal for child mortality, increased the rate of antenatal care five-fold, and increased the rate of postnatal care four-fold [8, 15–18]. However, national statistics and ongoing Demographic and Health Surveys (DHS) findings indicated there was no reduction in neonatal mortality, likely due to CHPS’s focus on community-based primary health care rather than on the rapid delivery of life-saving clinical interventions. During the GEHIP project, which began in 2011, the Upper East Regional Health Administration (UERHA) sought to identify and address service gaps for neonates at multiple levels of the health system, from CHPS in the community, to the sub-district, district, and regional levels.

#### Interventions

The GEHIP project objectives were accomplished through targeted audits, midwife training, and the development of clinical skills among community nurses. First, participatory feedback of information was derived from a program of targeted audits: death audits of all maternal and neonatal deaths that occurred in facilities in the region, and a 2011 audit of health system referrals of mothers and neonates [19]. The maternal and neonatal death audits are qualitative narratives describing the complete circumstances leading to each death. Maternal and neonatal death audits were conducted at the facility or sub-district in which they occurred, reviewed on a weekly basis at the regional level, and presented to all District Health Management Teams in the region at a monthly conference.

The GEHIP program trained midwives at the sub-district and village levels in neonatal resuscitation using a bag and mask to prevent birth asphyxia [20–24]. GEHIP implemented a policy of consistent partograph [25–27] use and clinical trainings to support this new policy, given identified challenges to proper use [25, 28, 29]. Third, GEHIP provided CHPS community nurses (Community Health Officers, or CHOs) who faced obstetric emergencies with training in essential clinical skills in emergency delivery and instituted a program to upgrade CHPS nurse training to midwife status. The final clinical skill added by GEHIP was training in community newborn care for CHO nurses and community volunteers, an evidence-based task-shifting strategy [30–32]. Furthermore, the UERHA supported the development of a sustainable emergency referral and transport system for remote CHPS zones, known as Sustainable Emergency Referral Care. This program guaranteed free transport for pregnant women and children in health emergencies and supported emergency referral of neonates and pregnant women.

#### Evaluation methods

Overall evaluation of outcomes was through the baseline and endline population health surveys, in combination with the DHS data and facility process data. The project team looked at change in neonatal mortality over time and process measures for the delivery of the intervention.

#### Implementation outcomes

The audits brought to the attention of senior management key challenges to neonatal survival, such as the high number of deaths due to birth asphyxia and processes contributing to asphyxia. Informational feedback involved UERHA leadership and health staff at all levels in problem solving and led to the development of an enhanced neonatal survival intervention package implemented by GEHIP at all levels of the health system (Table 3). Correct partograph training led to an increase in correct use. The training in community newborn care for CHO nurses and community volunteers resulted in improvements in the process of care in neonatal resuscitation/asphyxia management and kangaroo mother care. Some of the challenges of the implementation included getting the trainers and experts from outside the region to build capacity for neonatal care. Additionally, costs, and obtaining enough staff at the facilities have been highlighted as new areas for improvement.

**Table 3:** GEHIP participatory information feedback and clinical training interventions in Ghana PHIT intervention areas

Activity	Description
Audits for Information Feedback	• Audit of referral system for pregnant or recently pregnant women and newborns in 16 facilities over a six-month period in 2011; midwife-led teams tracked 446 referred women until receipt of definitive treatment. • Maternal and newborn death audits conducted for every newborn and obstetric death in region; monthly feedback to all levels of the health system.
Sustainable Emergency Referral Care	• Emergency referral and transport system for remote CHPS zones • Ambulances and trained volunteer drivers from the community work closely with CHPS nurses
Emergency Delivery Skills for Frontline Community Health Workers	• 60 CHOs placed in maternity and delivery wards to observe deliveries and enhance their confidence in managing emergency deliveries
Neonatal Resuscitation	• All frontline workers equipped and trained in provision of neonatal resuscitation using the Helping Babies Breathe curriculum. • Regional trainers and district nurse supervisors trained in neonatal resuscitation to support long-term sustainability. • Newly qualified and existing midwives trained annually in neonatal resuscitation.
Kangaroo Mother Care	• Newly qualified and existing CHPS midwives trained annually in kangaroo mother care.
Proper Management of the Sick Newborn	• CHOs, Physician Assistants, midwives, and staff nurses trained in facility and community-based newborn care. • Emphasis on postnatal visits on day 6 or 7 including assessment of the neonate for danger signs, treatment, referral, and counseling on child care practices.

### Performance review and enhancement: strengthening integrated primary health care in Sofala Province, Mozambique

#### Implementation objectives

The Mozambique PHIT strategy to improve the delivery of newborn care had two main objectives: 1) improved data use and analysis, and 2) strengthened capacity in health system management. These were combined through district performance review and enhancement meetings as an approach to use data to motivate quality improvement activities. These meetings brought together front-line health workers with district and provincial managers to review key performance indicators, identify gaps in performance, plan solutions, and monitor the implementation of these action plans.

#### Interventions

Beginning with their initial pilot in 2012, these cycles have become a cornerstone of the Mozambique PHIT project’s implementation strategy, as they effectively bridge data quality assessment and improvement efforts [11, 33, 34], while stimulating data-driven decision-making to lead to better management decisions at the facility, district, and provincial levels. They have also been extremely popular with health system partners, and continue to expand to more program and support areas. The district performance review and enhancement meetings first began in 2012 with maternal and child health (MCH) staff, and by 2015 have been expanded to include pharmacy, malaria, and tuberculosis meetings.

Part of the success of these meetings was that they were co-organized between the PHIT project staff and the local Provincial Health Department, and participants included Ministry of Health (MOH) paid and supported managers and front-line clinical providers. MOH leaders of the program for maternal and child health led decision-making on how to best conduct the meetings, provided feedback on the action plans, and led supervision efforts after action planning. This increased acceptability, uptake, and attendance of provincial, district, and health facility representatives, as this was seen as a core duty for their position as health system managers.

#### Evaluation methods

During these meetings, which were planned to occur at least every 6 months, health facility leadership presented and reviewed secular trends in outputs and coverage estimates across a range of MCH services, including reproductive health (antenatal care to birth to postpartum care/family planning) and child health. Meetings were jointly organized between provincial and district MCH leadership, along with technical support from Health Alliance International field staff. The district MCH director or the Chief Medical Officer for the district facilitated meetings. Four days prior to the meetings, district MCH supervisors, along with leadership and Health Alliance International technical staff, reviewed data and presentations, and provided mentorship around data interpretation and presentation. At the meetings, action plans were developed and updated based on new data trends to reflect priority activities and timelines to respond to identified problems. MCH nurses at each health facility worked with district leadership to develop and implement feasible action plans. Implementation of previous action plans was shared at subsequent meetings, facilitating accountability and peer learning across facilities of commonly shared challenges and spread of successful interventions.

#### Implementation outcomes

Challenges related to the performance review and enhancement meetings centered on monitoring and evaluation of targeted areas for improvement, tracking of action plans over time and across multiple meetings, and standardization of evaluation components for the program. Given the myriad different problems identified (see Table 4), spanning stock-outs of essential supplies, infrastructure needs, lack of appropriate knowledge, and concerns around health information system accuracy, it was not a simple process to evaluate whether action plans were indeed carried out, or if problems were resolved. An additional challenge was turnover of personnel at health facilities. Given that meetings occurred every 6 months, the high level of staff turnover – especially at rural clinics where staff were often assigned to do their short-term service – presented challenges to follow-up on action plans proposed at the previous meeting. As this program continues, the team is focusing on increasing supportive supervision and action plan follow-up between meetings, and creating simple yet effective action plan decision-support tools to both track progress towards action plans and provide more robust analyses of program effects.

**Table 4:** Sample action plans around maternal child health issues developed at district performance review and enhancement meetings in Sofala, Mozambique

Location at health center or hospital	Problem identified	Immediate action plan
Antenatal Care	Few women receiving malaria prophylaxis.	Re-train health care workers on criteria for when to offer malaria prophylaxis to pregnant women.
	Low knowledge of criteria used to administer co-trimoxazole to HIV-exposed pregnant women.	Re-train health works in staging and interpretation of prophylactic algorithm.
	Additional room needed for ANC visits to facilitate completion of all necessary tests and procedures.	Advocate to district chief medical office to have 2 rooms available for ANC visits.
	Stock-outs of mosquito nets.	Advocate to the district health office to provide health facility sufficient stock to guarantee a buffer.
Delivery Services	Low capacity to diagnose complicated obstetric cases.	Improve diagnosis of obstetric complications via discussion of cases and practice in the delivery room. Biweekly review partographs with health facility team.
	High number of premature/low birthweight births registered in previous period.	Verify accuracy of data by checking the scales in the health facilities for accuracy; if not, purchase new ones.

Sample action plans are included in Table 4, including issues related to quality of care, training needs, and systems gaps, such as stock-outs. In response to comments from site visits, a post-meeting monitoring tool was developed to document the implementation of action plans and the impact of these changes.

### Integrated clinical and system level improvement: the All Babies Count initiative in southern Kayonza and Kirehe districts, Rwanda

#### Implementation objectives

The Rwanda PHIT project used a health system strengthening approach to improve access and quality of care at all system levels in maternal and child health [9]. When examining progress in child health, the Rwanda PHIT project made impressive strides in reducing child deaths; yet, by 2013, mortality during the neonatal period had not seen the same progress. To that end, Partners In Health, together with the Rwanda Ministry of Health, created the All Babies Count (ABC) initiative, launched in October 2013 in 25 health facilities in southern Kayonza and Kirehe districts, serving a population of approximately 480,000 people [35].

The approach was designed as a platform to improve the delivery of life-saving interventions through pregnancy, delivery, and the postnatal period using a district-wide, multi-level approach. The design built upon clinical mentorship strategies that had previously seen improvement in broader maternal and child health outcomes, to add a strengthened system-level quality improvement component [36, 37]. At inception, utilization of facility-based delivery was relatively high, but formative programmatic data indicated that coverage and quality of key services delivered remained a key gap (Table 5) [38]. Additional national surveillance data indicated that facility delivery rates were as high as 80–90% in the planned intervention area. Therefore, upon agreement with the Rwanda Ministry of Health, ABC was created as an intensive 18-month change acceleration process to increase health system capacity to prevent newborn deaths by improving the quality and coverage of antenatal care, delivery management, and postnatal care in the newborn care continuum.

#### Interventions

**Fig. 2:**
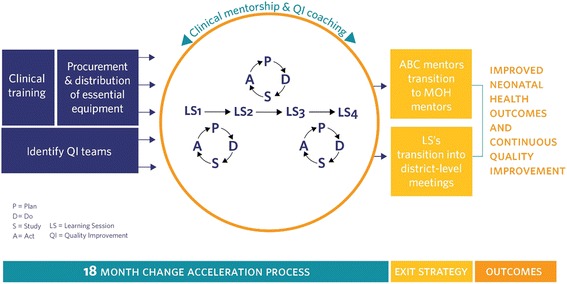
The All Babies Count Initiative intervention components

The ABC model, instead of focusing on a single intervention, worked to create sustainable impact by re-orienting the culture of the health system to one of continuous quality improvement (QI). This approach: 1) equipped health facilities by filling gaps in essential equipment and materials for neonatal care identified through a structured assessment process; 2) provided training and on-site, regular clinical mentorship to improve delivery of evidence-based interventions; and 3) launched district-wide learning collaboratives to build healthcare workers’ leadership in data utilization for continuous QI. The factors contributing to newborn deaths occur across pregnancy, delivery, and postnatal periods, as well as multiple levels of the health system. This combination of clinical mentorship and health system strengthening aimed to address the multi-causal nature of neonatal mortality and overcome identified weaknesses of vertical, fragmented interventions while addressing multiple points along the maternal newborn continuum of care [39, 40] (see Fig. 2).

To start, program leaders and ABC mentors provided maternal and neonatal care providers in each district with a newborn care training package along with the essential materials needed to implement quality care. Health facilities then identified interdisciplinary QI teams with members across the health system levels (community health supervisors, data officers, health center nurses, and hospital teams). During a series of four to five mentor-led learning sessions in each district, QI teams met to review performance, identify specific process barriers, and innovate, implement, and test ideas to improve quality of care. Teams used standard plan-do-study-act QI cycles, which were supported by ABC mentors, targeted key indicators in the neonatal care continuum—antenatal care, delivery, and post-partum care—and worked to build leadership skills.

#### Evaluation methods

The ABC intervention formally began with the first set of baseline trainings and a learning session in September 2013. Targeted process indicators focused on the antenatal, intrapartum, and postnatal periods. These data were derived from routinely collected mentor tools. Indicators derived from the health management information system were validated by register review and corrected as needed. Data for newly introduced indicators were derived from registers introduced to facilities at the start of the program period.

#### Implementation outcomes

At 18 months of implementation, health facilities had received an average of one visit per month by the clinical/QI mentor, and all health facilities had participated in each of the five learning sessions. All health facilities maintained at least two nurses trained in essential newborn care, and 60 QI projects were being implemented by facility teams across the districts. Over the 18-month learning collaborative period, improvement in targeted process indicators in the antenatal, intrapartum, and postnatal periods was seen (Table 5).

**Table 5:** All Babies Count initiative program monitoring data of key newborn care processes, Rwanda

Indicator	Baseline Sep-Oct 2013 number (%)	18 months Apr-Jun 2015 number (%)
4-visit ANC	807/3494 (23)	1362/3585 (38)
Early ANC registration	986/4201 (23)	1445/4250 (34)
Percent of women with pPROM treated with antibiotics	5/21 (24)	8/21 (38)
Percent of women with preterm labor who are treated with antenatal corticosteroids	5/19 (26)	18/24 (75)
Facility Delivery	2745/3155 (87)	4635/4879 (95)
^a^Time to C-section in minutes (median, [IQR])	99 [50–195]	72 [59–77]
Immediate skin-to-skin care after delivery	100/523 (19)	3719/4275 (87)
Newborns checked for danger signs within 24 h of birth	1640/3489 (47)	3671/3746 (98)

^a^Southern Kayonza only: BL (*n* = 35), 18mo (*n* = 18)

**Table 6:** Baseline country characteristics of factors related to neonatal mortality^a^

	Tanzania^b^	Zambia^c^	Ghana^d^	Mozambique^e^	Rwanda^f^
Total Fertility rate (number, 95% CI)	4.6 (4.4–4.8)	5.7 (5.4–5.9)	5.0 (4.7–5.3)	7.5 (6.1–8.9)	5.1 (4.8–5.4)
Contraceptive Prevalence (percent, 95% CI)	36.3 (33.9–38.8)	50.2 (48.2–52.3)	14.2 (12.9–15.5)	43.34 (36.0–51.0)	46.2 (42.5–49.9)
Antenatal Care 4+ Visits (percent, 95% CI)	42.9 (38.2–47.9)	50.2 (45.7–4.7)	85.7 (83.8–87.7)	Not available	27.4 (23.8–31.0)
At least 1 Antenatal Care Visit (percent, 95% CI)	98.9 (97.9–99.9)	92 (81.6–99.1)	93.9 (92.6–95.2)	93.6 (86.0–97)	98.3 (97.5–99.2)
Skilled Attendant at Birth (percent, 95% CI)	73.6 (69.4–77.7)	72 (32.5–83.1)	54.0 (51.2–56.9)	55.0 (52.2–58.3)	64.6 (60.4–68.8)
Postnatal Care for Mother (percent, 95% CI)	30.28 (25.9–34.7)	5.4 (3.3–8.3)	Not available	Not available	17.2 (13.3–21)
Neonatal Mortality (deaths/1000 live births, 95% CI)	23.6 (21.4–26.1)	24 (13.5–41.2)	16 (Not available)	30.4 (27.3, 33.8)	27.7 (16.6–38.9)

^a^All data come from the intervention area estimates in the AHI baseline metrics except for Zambia, which only reported non-intervention area estimates

^b^Total fertility and neonatal mortality from Ifakara and Rufiji Health and Demographic Surveillance System (HDSS) (2007–2011) and contraceptive prevalence, antenatal care, skilled attendant at birth, and postnatal care indicators from baseline household survey (2009–2011)

^c^Data from 2013 Zambia Demographic and Health Survey except for antenatal care 4+ coverage and postnatal care for mother, which were extracted from 2012 program survey data

^d^Data from 2011 Program Baseline Household Survey

^e^Data from 2008 Multi-indicator Cluster Survey (MICS) except for neonatal mortality, which was based on combined analysis of 2011 Mozambique Demographic and Health Survey and 2008 MICS

^f^Data from 2010 Rwanda Demographic and Health Survey

While there were a number of implementation successes, significant challenges had to be addressed throughout the intervention period, and the design was adapted to optimize impact. For example, after observing that quality improvement activities took longer for rural facility teams to engage in than anticipated, the learning collaborative length was increased from 12 to 18 months, coaching was intensified, and health system leaders were more strongly engaged in learning session leadership and participation. Additionally, some QI teams found that their leadership was reluctant to support activities that they were not technically familiar with themselves; QI trainings were therefore offered to health facility and district leadership to build their comfort with and understanding of their role in catalyzing improvement. Furthermore, existing MOH supervisors were engaged throughout the collaborative period as co-mentors with the ABC mentors to build their capacity to sustain and deepen improvement moving beyond the collaborative period. However, staff turnover of these supervisors occurred towards the end of the collaborative, which served as a challenge to long-term capacity building at the mentor level. A sustainability analysis is underway to evaluate the durability of improvements achieved 1 year after the end of the formal collaborative.

### Cross-site results

Across the PHIT projects, there were a number of commonalities, differences, and lessons learned in the strategies designed and implemented to improve newborn care and health. While each country emphasized the different themes described above, all included components focused on supporting at least some, if not all, of the WHO Health Systems Framework building blocks to improve systems of care for vulnerable newborns (Table 7).

**Table 7:** PHIT crosscutting strategies across countries using WHO health system building blocks

World Health Organization Health System Building Block	Tanzania	Zambia	Ghana	Mozambique	Rwanda
Service Delivery	Community health worker training	Clinical training and supportive supervision, partograph introduction	Clinical training, sustainable emergency referral care	Action planning focused on addressing quality gaps	Clinical training sustained with on-site mentorship
Health Workforce	Strengthened front-line community health workers	Strengthened community and facility health workers	Implemented program to upgrade community health nurses to nurse midwives	Training on data interpretation and review; improved data-driven supervision structures	Supported management and organization of staff through quality improvement efforts
Information and Research	Data sharing annually	Electronic medical record introduction, support for data feedback at community and facility levels	Death audits, referral audit study and feedback loops	Data feedback and action planning	Data feedback, gap identification, quality improvement implementation and data monitoring
Medical Products and Technologies	Ambulance support, cell phone provision to key health care providers	Provision of key equipment for complete antenatal and postnatal assessment	Neonatal resuscitation equipment provision	Action planning on how to ensure availability of essential commodities	Provision of essential package of equipment
Health Care Financing	Decreased cost through task-shifting	Support for TBAs and community health workers with a monthly stipend	Catalytic financing of $0.85 per capita per year	Funds to support action plan implementation	Some QI projects focused on cost reduction/insurance enrollment if identified as barrier to services
Leadership/ Governance		Training for District Medical Officers in Health Management	Advocacy with district political systems to improve health care financing and catalyze support for expanding coverage of primary health care	Strengthened management and data driven decision-making by district leadership	Staff motivation and leadership development through quality improvement activities

When PHIT projects were analyzed using a continuum of care framework (Table 8), some differences and commonalities emerged in how these blocks were strengthened. Tanzania and Zambia included significant community-based components, including mobilization and sensitization for increased uptake of essential services, while Ghana, Mozambique, and Rwanda focused more efforts on improving the quality of services delivered once a patient enters a health facility. All countries included aspects that improved communication across levels of the health system, whether through district-wide data sharing and peer learning networks in Mozambique and Rwanda, or improved referral processes and systems in Tanzania, Zambia, and Ghana.

**Table 8:** Country interventions along the continuum of care

	Household and community	Front-line outpatient facility	Hospital	District
Pregnancy	Tanzania, Zambia	Ghana, Rwanda, Zambia	Ghana, Rwanda	Ghana, Mozambique, Rwanda, Tanzania
Birth	Ghana, Tanzania, Zambia	Ghana, Rwanda, Zambia	Ghana, Rwanda	Ghana, Mozambique, Rwanda, Tanzania
Postnatal	Ghana, Tanzania, Zambia	Ghana, Rwanda, Zambia	Ghana, Rwanda	Ghana, Mozambique, Rwanda, Tanzania

## Discussion

There were a number of key lessons learned based on these experiences. First, all PHIT projects found that an intentional focus on improving care delivery by targeting drivers of neonatal deaths and reflecting local context was critical to improving newborn health and outcomes. Each PHIT project appropriately adapted their intervention to reflect baseline needs, local context, and government priorities, using the resources available. The All Babies Count initiative in Rwanda was a focused neonatal quality of care improvement initiative across the neonatal care continuum and at all facility levels; however, they did not have a large focus on demand creation, reflecting results from the formative assessment and baseline high facility delivery rate. The Zambia PHIT project designed their neonatal work partway into the implementation time period, after finding that they were not seeing the desired neonatal health improvements using their more general quality improvement approach in routine outpatient service strengthening.

Second, given the complex nature of the drivers of neonatal health outcomes—occurring at multiple time points from pregnancy through delivery and postnatal care, and at all system levels—we found that consistent with the continuum of care framework [5, 41–44], improvement in newborn care and outcomes requires complex and integrated health system approaches, rather than narrow, vertical interventions. For example, the ABC initiative was developed as an integrated approach targeting key drivers of improvement in the quality of essential maternal and newborn services: a strong health system that develops leaders who support the use of proven care practices, a culture of improvement, and strengthened individual provider skills and motivation in key newborn care processes. The Zambia PHIT project focused on both demand creation and supply-side interventions, and GEHIP in Ghana designed interventions at all levels of the health system to address identified gaps in quality and coordination of care. Interestingly, the Tanzania Connect project made some course correction in the implementation of interventions to improve newborn health, but did not see positive impact on maternal newborn care process measures in the intervention area. Possible reasons could be that the interventions were aimed only at service utilization with a community-based approach, and were not as comprehensive as other initiatives in their system approach, without inclusion of community case management, or service quality improvement at multiple levels.

Third, we found that encouraging utilization of services must be met with high quality services. The Tanzania Connect project focused its efforts entirely at the community level (care and referrals). However, this approach assumed that health facilities were adequately prepared to care for these complicated cases. While these efforts improved the rates of emergency referrals, they were not sufficient to markedly reduce the rate of neonatal deaths (Almamy Malick Kante, PhD, MSc, 2015, oral comm., October). Similar to other studies, they found that the burden of newborn deaths shifted from the community to the health facility, as most health facilities were unprepared to adequately treat newborns with serious complications [3, 45–47].

Finally, leadership engagement and ownership was critical to interventions aimed at changing the culture of care provision. In the Mozambique PHIT project, district performance review and enhancement meetings were led by district-level public sector staff, helping to create a culture of data review and data-driven decision-making. The Rwanda ABC initiative relied on leaders’ ownership of the initiative at the central, district, and facility levels as critical to programmatic success, since the learning collaborative approach and site-level coaching required high participation and engagement in order to create change.

Many of the PHIT projects encountered similar implementation challenges to feasibility and ensuring fidelity to the strategies including staff turnover, leader ownership of technical programs to support sustainability of improvements gained, shortage of system inputs such as equipment and medications, and data quality. For example, sustainability of these improvements in the face of MOH leadership and mentor turnover remains to be seen and is the subject of further investigation. Additionally, follow-through on action plans made was a common challenge across multiple programs including the Mozambique and Rwanda PHIT projects. Yet, all programs worked to address these challenges using the resources available with variable results. In three countries – Rwanda, Tanzania, and Ghana – broader policy impact is being witnessed, as some of the PHIT partners’ interventions have been adopted or expanded nationally. For example, in Rwanda, the partners have worked with the MOH to expand the ABC program to seven new districts, and over 50,000 additional women [48].

This analysis has some limitations. Creating cross-site metrics was a challenge given the variability in local data sources and definitions across country cases (despite common core indicators), and therefore limited the scope of baseline metrics presented for understanding individual project context. As each PHIT program included intervention elements related to neonatal health at different time points in their intervention based on their context and experience, differing intervention-specific evaluation strategies and timelines limited ability to present outcomes or impact data for most case studies. Additionally, measuring impact on neonatal mortality relates to vital events recording, which is limited by the lack of birth/death registration in many resource-limited settings. Additionally, case study methodology can have limits to generalizability, as it involves an in-depth look at a small number of examples. However, using the combination of the WHO building block of health systems framework and the maternal newborn child health continuum of care framework allows for extraction of key similarities and differences to distill critical lessons that could be of relevance to implementers, public health practitioners, donors, and local and global policy makers when considering how to best positively impact newborn health through a health system strengthening approach.

## Conclusion

The PHIT projects across five countries all employed strategies to improve newborn health, an area where it has been difficult to create change, using multifaceted integrated health system approaches. These diverse experiences, while adapted to each implementation setting and challenges encountered, shared a common theme of integration—integration across health system levels with an emphasis on leadership development, and across the maternal and newborn care continuum.

## Abbreviations

ABC: All Babies Count initiative
AHI: African Health Initiative
ANC: Antenatal care
BHOMA: Better Health Outcomes through Mentoring and Assessment
CHA: Community Health Agent
CHO: Community Health Officer
CHPS: Community-Based Health Planning and Services program
DDCF: Doris Duke Charitable Foundation
DHS: Demographic and Health Survey
GEHIP: Ghana Essential Health Intervention Program
IMCI: Integrated Management of Childhood Illness
MCH: Maternal and child health
MOH: Ministry of Health
PHIT: Population Health Implementation and Training partnerships
PPS: Probability Proportional to Size sampling
QI: Quality improvement
TBA: Traditional Birthing Attendant
UERHA: Upper East Regional Health Administration
WHO: World Health Organization
